# Gonorrhœa and Homœopathy*

**Published:** 1890-12

**Authors:** T. F. Allen


### GONORRHŒA AND HOMŒOPATHY.*

EDITORS OF THE HOMŒOPATHIC PHYSICIAN :

So much bitterness has been shown on account of my opinion concerning the treatment of some cases of gonorrhœa and so many adjectives and epithets have been put into your pages that I venture, very timidly, to say a few words, though I suppose it were better to keep still and follow my own way in peace, a course I shortly intend doing, both publicly and privately. Is it not possible for physicians to respect the opinions of others and refrain from unpleasant appellations or must every one be made a target of? But that rests chiefly with the editors of our journals and I will say no more about it.

1st. Observing a number of patients afflicted with gonorrhœa to run a tedious course under purely homœopathic treatment (one man suffering eighteen months continuously though treated by a careful prescriber with the two-hundredth dilution); observing, farther, that physicians practicing pure Homœopathy in such cases treated few cases and still fewer as the years passed on, I have endeavored to ascertain the reason for this lack of success.

2d. Observing that gonorrhœa, syphilis, hydrophobia, etc., attack persons in apparently perfect health and also observing that these patients require remedies directed to the immediate effects of the poison rather than to the individual dyscrasia, which permits a *disease* to develop and continue, I have con-

---

*See Dr. Thomas Skinner's article, "The Routine Treatment of Fresh Gonorrhœa," in November number of this journal, page 498.

cluded that gonorrhœa may be considered a *poison* rather than a disease, and hence may be treated as such rather than homœopathically. The best method of treating the local manifestations of this poison is a matter of experience. Some cases will last but a few days, others months. A few cases will develop constitutional complications, others none. The suppression by violent measures of the discharge in the acute inflammatory stage may or may not be followed by grave remote effects. I have known a recurring iritis persisting twenty years to disappear only on the return of a purulent discharge from the urethra. I have known the kidney to become involved in a suppurative process and death averted only by emptying the pus through a drainage-tube in the back ; a host of bad things follow the injudicious treatment of the acute stage ; still, most satisfactory results do follow the persistent washing out the urethra with tepid water, to which a little Salt of Soda or Zinc or Mercury may be added, particularly after the first few days. A homœopathic physician who determines to practice only Homœopathy must send away a great many patients to other doctors if he would do the best for them. Men *will not* tolerate a discharge for months when a safe washing will help them get well speedily. Now another one will get well in ten days, but such are rare cases. It is unnecessary to risk stricture, cystitis, nephritis, orchitis, rheumatism, etc., by properly washing out the discharge. What is the harm ! ! We cleanse sores on the surface, we may be obliged to cleanse mucous surfaces to heal them. One may still be a consistent homœopathist and wash out or antidote by other means a gonorrhœal virus. Let us hear the other side, gentlemen ! but let us have a friendly discussion or none at all.

T. F. ALLEN.

